# Air quality and health co-benefits of climate change mitigation and adaptation actions by 2030: an interdisciplinary modeling study in Ahmedabad, India

**DOI:** 10.1088/2752-5309/aca7d8

**Published:** 2023-03-01

**Authors:** Vijay S Limaye, Akhilesh Magal, Jaykumar Joshi, Sujit Maji, Priya Dutta, Prashant Rajput, Shyam Pingle, Prima Madan, Polash Mukerjee, Shahana Bano, Gufran Beig, Dileep Mavalankar, Anjali Jaiswal, Kim Knowlton

**Affiliations:** 1 Natural Resources Defense Council 40 West 20th Street, New York, NY 10011, United States of America; 2 Gujarat Energy and Research Management Institute (Former), PDPU Road, Gandhinagar, Gujarat, 382007, India; 3 Indian Institute of Tropical Meteorology, Ministry of Earth Sciences, Dr Homi Bhabha Road, Panchawati, Pashan, Pune, Maharashtra 411008, India; 4 Indian Institute of Public Health, Gandhinagar, NH-147, Palaj Village, Gandhinagar, Gujarat 382042, India; 5 Mailman School of Public Health, Columbia University, 722 W 168th Street, New York, NY 10032, United States of America

**Keywords:** India, mitigation, adaptation, health, climate change, co-benefits, air pollution

## Abstract

Climate change-driven temperature increases worsen air quality in places where coal combustion powers electricity for air conditioning. Climate solutions that substitute clean and renewable energy in place of polluting coal and promote adaptation to warming through reflective cool roofs can reduce cooling energy demand in buildings, lower power sector carbon emissions, and improve air quality and health. We investigate the air quality and health co-benefits of climate solutions in Ahmedabad, India—a city where air pollution levels exceed national health-based standards—through an interdisciplinary modeling approach. Using a 2018 baseline, we quantify changes in fine particulate matter (PM_2.5_) air pollution and all-cause mortality in 2030 from increasing renewable energy use (mitigation) and expanding Ahmedabad’s cool roofs heat resilience program (adaptation). We apply local demographic and health data and compare a 2030 mitigation and adaptation (M&A) scenario to a 2030 business-as-usual (BAU) scenario (without climate change response actions), each relative to 2018 pollution levels. We estimate that the 2030 BAU scenario results in an increase of PM_2.5_ air pollution of 4.13 *µ*g m^−3^ from 2018 compared to a 0.11 *µ*g m^−3^ decline from 2018 under the 2030 M&A scenario. Reduced PM_2.5_ air pollution under 2030 M&A results in 1216–1414 fewer premature all-cause deaths annually compared to 2030 BAU. Achievement of National Clean Air Programme, National Ambient Air Quality Standards, or World Health Organization annual PM_2.5_ Air Quality Guideline targets in 2030 results in up to 6510, 9047, or 17 369 fewer annual deaths, respectively, relative to 2030 BAU. This comprehensive modeling method is adaptable to estimate local air quality and health co-benefits in other settings by integrating climate, energy, cooling, land cover, air pollution, and health data. Our findings demonstrate that city-level climate change response policies can achieve substantial air quality and health co-benefits. Such work can inform public discourse on the near-term health benefits of mitigation and adaptation.

## 1. Introduction

Air pollution is a major global public health problem, associated with an estimated 6.67 million deaths in 2019 from outdoor and indoor exposures combined, of which 4.2 million are due to ambient (outdoor) air pollution exposures (Health Effects Institute 2020). Fine particulate matter (PM_2.5_, with aerodynamic diameter ⩽2.5 *µ*m) poses severe health risks via penetration of particles deep into the lungs and into the bloodstream, resulting in acute and chronic cardiovascular and respiratory impacts, including lung cancer and cerebrovascular ailments (Health Effects Institute 2020). While global average PM_2.5_ levels declined slightly from 2010 to 2019, concentrations in South Asia worsened during that period and reflect the highest exposures globally (Health Effects Institute 2020).

In India, outdoor air pollution imposes an enormous public health burden, contributing to an estimated 980 000 deaths in 2019, when the country’s annual PM_2.5_ level averaged 91.7 *µ*g m^−3^ (Pandey et al 2020). Average annual exposures across many Indian cities are well above the current Indian National Ambient Air Quality Standard (NAAQS) (annual average of 40 *µ*g m^−3^) and the corresponding World Health Organization Air Quality Guideline (WHO AQG) (annual average of 5 *µ*g m^−3^) (Balakrishnan *et al* 2019, Purohit *et al* 2019, Pandey *et al* 2020, World Health Organization 2021). Importantly, premature mortality from air pollution in the 12 current Indian ‘megacities’ (population >10 million, including Ahmedabad, Mumbai, Pune, and Kolkata) increased significantly between 2005 and 2018 (Vohra *et al* 2022). That change stems in part from sulfur dioxide (SO_2_) emissions (a contributor to secondary PM_2.5_ formation in the atmosphere) from industry and coal-fired power plants that increased by 50% between 2007 and 2016 (Li *et al* 2017).

Climate change, air pollution, and extreme heat are interconnected public health threats: combustion of fossil fuels emits health-harming fine particles as well as carbon pollution that fuels rising temperatures. Extreme heat in India is already associated with significant excess all-cause mortality (Azhar Shah *et al* 2014), and climate change is projected to further increase annual average temperatures as much as 4.4 °C (∼8 °F) by the 2080s (Sanjay *et al* 2020) relative to the 1976–2005 average. Recent air modeling studies led by Indian researchers indicate that climate warming could worsen PM_2.5_ pollution across the country (Upadhyay *et al* 2020, Kaur and Pandey 2021).

Air conditioning (A/C) offers an important heat-health adaptation, as recognized in India’s Cooling Action Plan (ICAP). Although only 6% of Indian households were estimated to have A/C in 2019 (Romanello *et al* 2021), the ICAP projects nationwide cooling demand to grow eight-fold by 2037–38 compared to 2017–18 (Ministry of Environment, Forests, and Climate Change (Government of India) 2019a). The ICAP sets forth a comprehensive cross-sectoral plan to address India’s growing demand for cooling energy through climate-friendly solutions (Ministry of Environment, Forests, and Climate Change (Government of India) 2019a). In addition to planning for increased cooling needs, the Government of India also launched a National Clean Air Programme (NCAP) in 2019 to provide a roadmap for reducing unhealthy air pollution levels (Ganguly *et al* 2020). The NCAP aims to reduce PM_2.5_ levels 20%–30% by 2024 (relative to 2017) levels in 132 cities not yet attaining the annual NAAQS (CPCB 2021).

While A/C saves lives, its use can worsen both air pollution and climate change itself, if the energy to meet associated increases in electricity demand is supplied from fossil fuels (Abel *et al* 2018). India operates 47 thermal coal-fired power plants more than 25 years old, with an average age of 34 years (Singh and Sharma 2021, Global Energy Monitor 2022), including the torrent power plant (TPP) in Ahmedabad, opened in 1934. Prior work estimated that in India, air pollution from coal combustion in thermal power plants and industries contributes to 169 000 premature deaths annually (Ganguly *et al* 2020). Electricity use for A/C is becoming more sensitive to rising temperatures in India (Gupta 2012), with unclear health effects.

The city of Ahmedabad has developed coordinated climate resilience strategies to reduce health vulnerabilities from extreme heat and air pollution. Ahmedabad developed and launched South Asia’s first heat action plan in 2013, an effort integrating heat forecasting with improved municipal governance, health risk communication, and landcover interventions, including reflective cool roofs (Knowlton *et al* 2014, Pingle *et al* 2018). Cool roofs can help to moderate indoor temperatures and save energy from reducing A/C and fan use for cooling Vellingiri *et al* 2020. In 2017, Ahmedabad launched an air information response plan (Limaye *et al* 2018a) to establish continuous air quality monitoring and health risk communication.

This project characterizes the current state of local air quality and cooling demand in Ahmedabad and projects 2030 PM_2.5_ pollution levels and air pollution effects on all-cause mortality from implementing climate change mitigation and adaptation (M&A) strategies (compared to a business-as-usual (BAU) future). To do so, our project team conducted an interdisciplinary analysis spanning climate, energy, air pollution, and health models to estimate future air pollution and health co-benefits at the city level. We configure the Benefits Mapping and Analysis Program-Community Edition (BenMAP-CE) with local air pollution, population, and baseline health data to estimate, for the first time, citywide air quality effects on human health. Because this research is executed using an adaptable modeling framework and designed within the context of the ICAP, NCAP, and India’s climate goals, it can help to inform broader city and state actions across the country to address climate change and achieve near-term improvements for air quality and public health.

## 2. Methods

Recent guidance on ways to better standardize health co-benefits analyses suggests that researchers clearly describe their modeling approaches, methods for parameterization and reporting, and approaches for stakeholder engagement (Hess *et al* 2020). With these suggestions in mind, a summary of the methodological approach taken by the project team follows.

### 2.1. Overview of modeling approach

**Figure 1.**
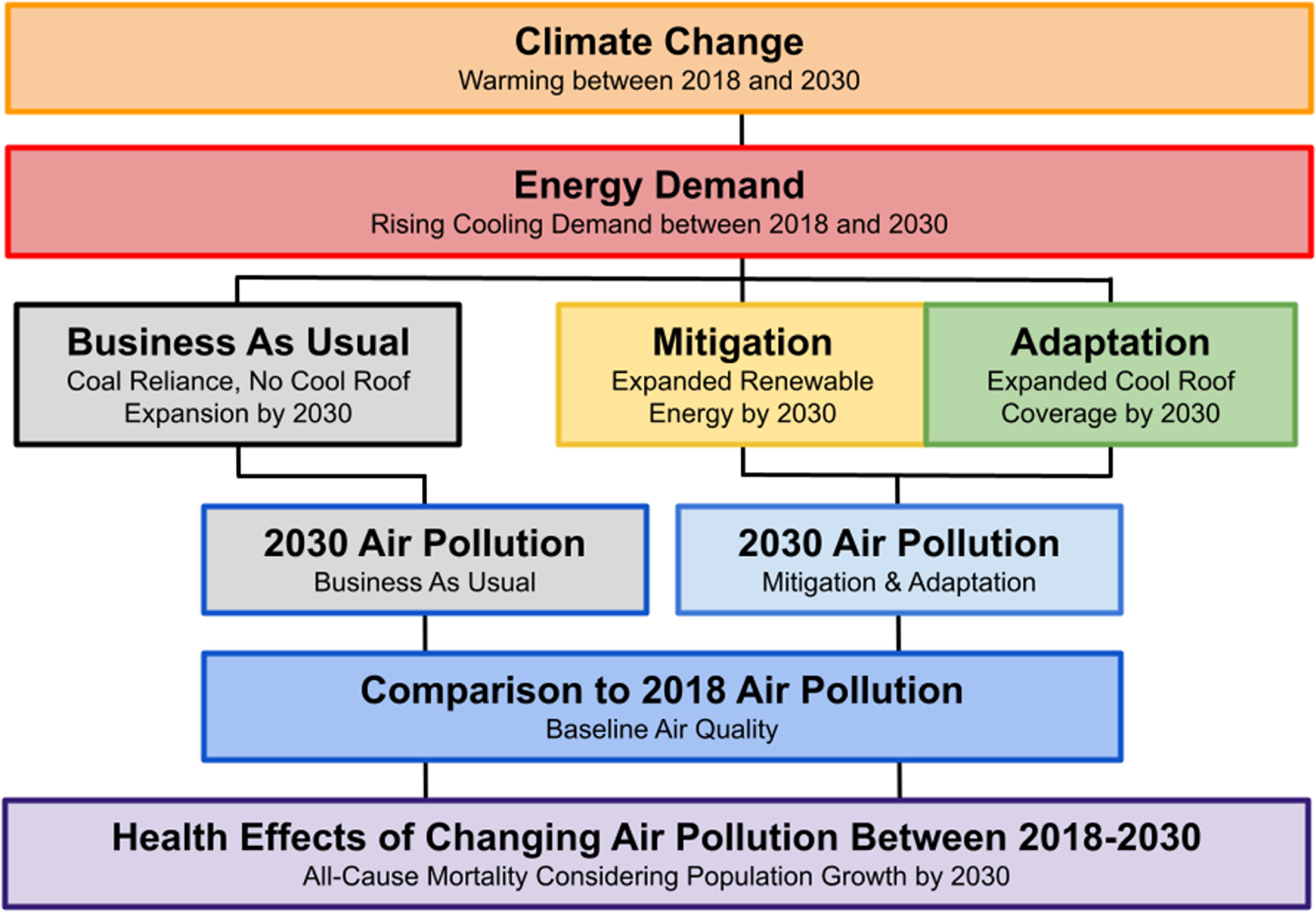
Flowchart depicting sequenced approach for air quality and health co-benefits modeling analysis. From top to bottom: climate change warming in Ahmedabad estimated between 2018 and 2030 under Representative Concentration Pathway 8.5, energy demand with a focus on electricity for cooling buildings, 2030 energy supply (coal-fired power plant under the business-as-usual scenario or renewable solar and wind energy under the mitigation scenario), and landcover modification via cool roof (adaptation scenario). Energy system futures are used to estimate 2030 air quality using the WRF-Chem model under two conditions: the business-as-usual (BAU) and combined mitigation and adaptation scenario (M&A). Air pollution effects on all-cause mortality in Ahmedabad are estimated using the BenMAP-CE model by comparing estimated 2030 air pollution levels (BAU and M&A) to baseline air pollution levels in 2018.

As shown in figure 1, to establish a baseline for analysis we first modeled demand for cooling energy in Ahmedabad in 2018 and the sources of energy supply (thermal coal-fired power plants or renewable sources including solar and wind energy) utilized to meet current electricity needs (sections 2.2–4). We then estimated electricity and cooling demand in 2030, considering changing demand for cooling driven by population growth, economic development, and climate warming (section 2.5). Energy modeling then informed the level of air pollution generated from thermal coal plant electric power delivery to Ahmedabad in baseline 2018 and in 2030, under a BAU future and a combined mitigation (energy source) and adaptation (land cover) scenario (section 2.6).

Air pollution modeling subsequently distributed the stationary energy source-generated air pollution emissions across the modeling domain, along with other regional air pollution inputs (section 2.6.1). Regional chemical inputs were pollutant concentrations of PM_2.5_ (and its precursor gases: sulfur dioxide, nitrogen oxides, volatile organic compounds, and primary particulate matter composed of dust, black carbon, and organic carbon) analyzed in a city-level domain nested in broader domain boundaries (see supplemental information section 1.3.3). Finally, associated changes in air pollution-related premature mortality, under the combined M&A scenario, were evaluated and compared using a health impact assessment model that integrates population, pollution exposure, and baseline health data with air pollution exposure-risk functions (figure 1 and section 2.6.2).

### 2.2. Geographic area

**Figure 2.**
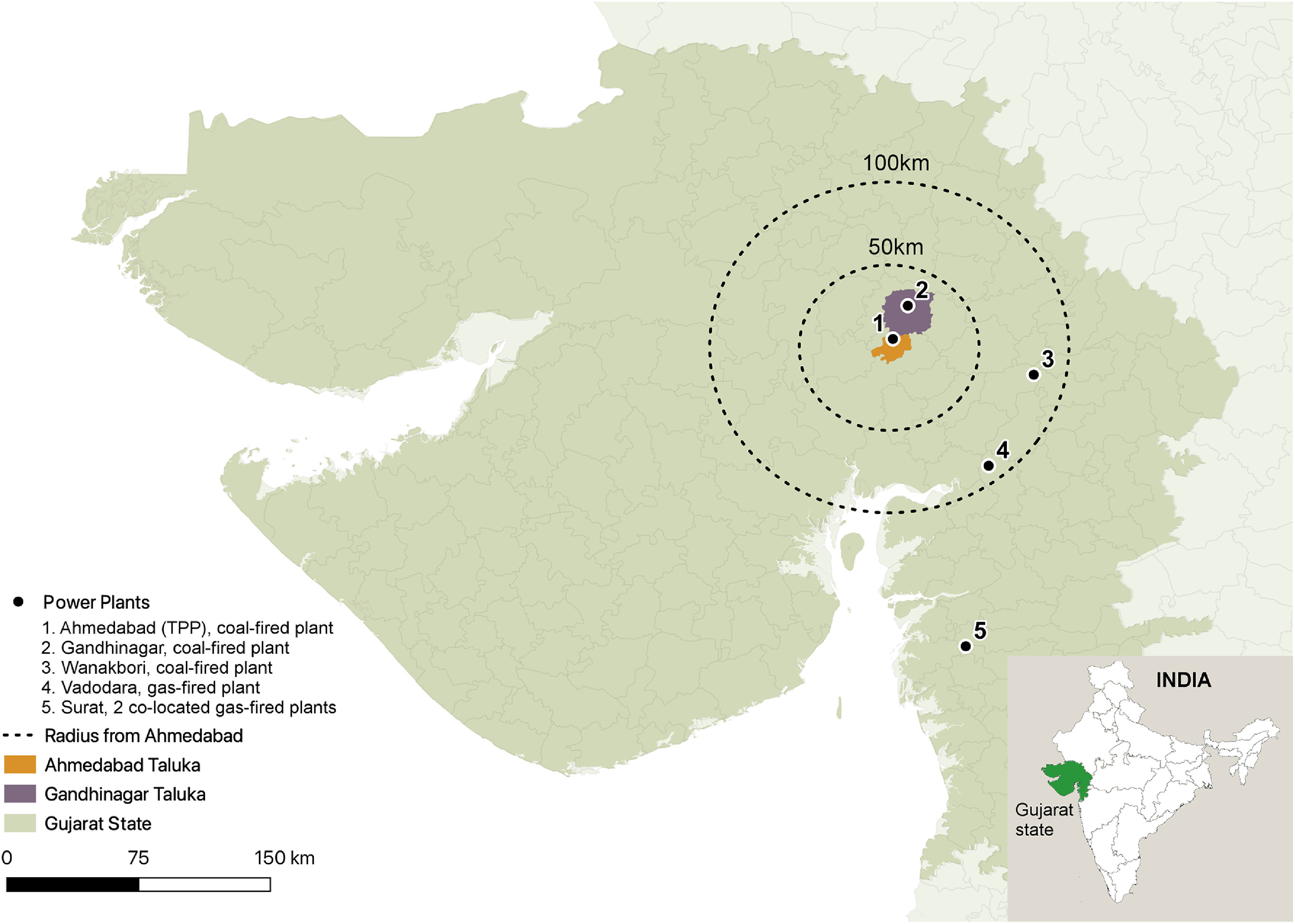
Map of Ahmedabad study area with inset map of in India and Gujarat state (green area). The study area map displays the six fossil fuel-fired power plants in Gujarat. The energy modeling analysis considers all plants depicted here, and air quality and health analyses focuses on the pollution impacts from the torrent power plant (TPP) in Ahmedabad (power plant #1). Adapted from (Joshi *et al* 2022), with permission from Springer Nature. CC BY 4.0.

This project focused on the municipal (taluka) area of Ahmedabad, a city of about 8.5 million people in Gujarat state in western India (see figure 2). While the urban area of Gandhinagar is immediately adjacent to the north and the two cities are often considered together, we limited the study to Ahmedabad because of availability of local population, baseline mortality, energy, and air pollution data. With respect to air pollution emissions sources relevant to air quality in Ahmedabad, there are two local thermal coal-fired power plants in the region, both relatively old, high pollution-emitting sources (see figure 2); there are six total fossil fuel-fired power plants in Gujarat (Gujarat Electricity Regulatory Commission (Gandhinagar) 2022b, p 147, (2022a), p 39, Joshi *et al* 2022).

As part of a related analysis (Joshi *et al* 2022), we identified major point sources of PM_2.5_ emissions that provide power to meet Ahmedabad’s energy demand and are in close enough proximity to also affect the city’s air quality (Guttikunda and Jawahar 2014, System of Air Quality and Weather Forecasting and Research, Indian Institute of Tropical Meteorology 2017, Ganguly *et al* 2020). The coal-fired TPP in the heart of the city on the Sabarmati River, opened in 1934, was the focus for modeling energy, air quality and health. TPP largely supplies municipal electricity and cooling needs, and TPP’s PM_2.5_ emissions directly impact the city’s air quality (Guttikunda and Jawahar 2014). Among the other power plants shown in figure 2, only the Surat gas-fired plants also supply power to Ahmedabad. However, as these plants are located approximately 200 km from the urban core, they do not appreciably impact city air quality.

The Gandhinagar Power Plant, while nearby, does not supply power to Ahmedabad, is already operating near capacity, and is projected to remain so in the foreseeable future (Joshi *et al* 2022), so its emissions were expected to remain fairly constant by 2030 and were considered as stable inputs to the overall modeling domain (Gujarat Electricity Regulatory Commission (Gandhinagar) 2020), see section 2.5.4. The Wanakbori and Vadodara plants, nearly 100 km distant, also do not supply power for Ahmedabad and thus their emissions are held constant in our analysis (Guttikunda and Jawahar 2014, Gujarat Industries Power Company Ltd 2016, Gujarat Electricity Regulatory Commission (Gandhinagar) 2020). According to India’s Central Electricity Authority, TPP is a ‘Category A’ power plant because of its location within a 10 km radius of a million-plus city (in this case, Ahmedabad); such plants are being prioritized for emissions controls to reduce pollution exposures to nearby population centers (Central Electricity Authority, Ministry of Power (Government of India) 2022).

### 2.3. Overview of baseline and future scenarios

We selected our analytical time frames of 2018 (baseline year) and 2030 as the model-simulated future year. Critical data on meteorology, energy demand, cool roof implementation, air quality, and citywide all-cause mortality were available and accessible for the year 2018. As the global coronavirus pandemic emerged in Asia in late 2019, spread globally in 2020 and 2021, and still continues, 2018 is among the most recent years for which data is available to represent a pre-pandemic reference. The year 2030 offers an opportunity for developing near-term air quality and health projections for local policymakers, who are engaged in planning air quality targets for 2024 under the NCAP and key national climate change goals (Intergovernmental Panel on Climate Change 2018). We describe the methods and assumptions for estimating annual average PM_2.5_ air pollution levels in the baseline and two 2030 scenarios in sections 2.4, 2.5 and table 1.

**Table 1.** Three air quality and health impact modeling scenarios applied (2018 baseline, 2030 business-as-usual (BAU), 2030 mitigation and adaptation (M&A)). TPP is the Torrent Power Plant in Ahmedabad.

Scenario	Energy sector emissions	Land cover adaptation
2018 baseline	• Direct estimate of TPP emissions in 2018 • 2018 meteorology and boundary conditions	• Estimates cooling energy demand from buildings consistent with 5% cool roof area coverage
2030 business-as-usual (BAU)	• Climate change affects ambient temperatures and cooling energy demand • 2030 meteorology and boundary conditions • TPP emissions reflect slight growth relative to 2018	• Estimates cooling energy demand from buildings consistent with 5% cool roof area coverage
2030 mitigation and adaptation (M&A)	• Climate change affects ambient temperatures and cooling energy demand (local application of India Cooling Action Plan estimates) • 2030 meteorology and boundary conditions • Eliminates TPP pollution emissions and assumes additional Ahmedabad city power demand met by renewable energy sources	• Assumes total of 20% cool roof area coverage (15% beyond the 5% baseline in 2018) leads to reduction in cooling energy demand from buildings

### 2.4. Estimating 2018 baseline

**Figure 3.**
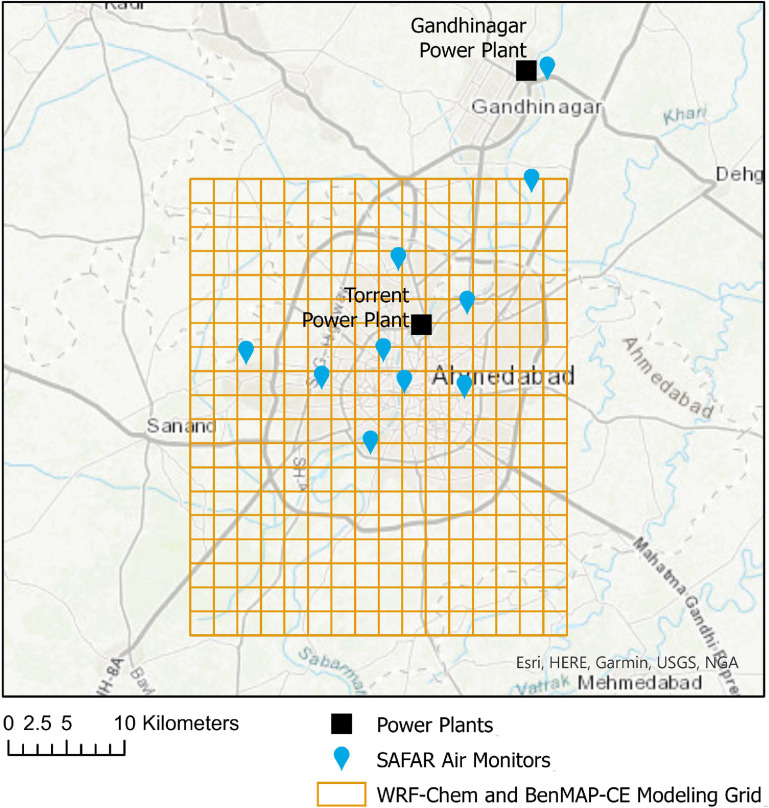
Map of study region with ten SAFAR (System of Air Quality and Weather Forecasting and Research) continuous air quality monitoring stations (blue pins), thermal coal-fired thermal power plants (black squares), and inner-outer grid domain (boundary) for WRF-Chem and BenMAP-CE modeling of Ahmedabad (orange square grid). For more information on the air quality modeling domain and baseline air quality estimates using monitored data, see supplemental information section 1.3. Figure produced using ArcGIS Pro (ESRI 2020).

We used the Weather Research and Forecasting-Chemistry model (WRF-Chem version 3.9.1) (Grell *et al* 2005, Powers *et al* 2017) to estimate 2018 meteorology and air pollution. To compare near-term future air pollution to 2018 pollution levels on a consistent geographic grid, we conducted an analysis in WRF-Chem of 2018 monitored and modeled conditions, using previously documented modeling approaches (Beig *et al* 2015, Limaye *et al* 2018a). We compared the use of modeled 2018 baseline data against 2018 daily ambient PM_2.5_ monitoring data collected from System of Air Quality and Weather Forecasting and Research (SAFAR) continuous air quality monitors (see figure 3 and supplemental table B).

We compared the ramifications for health effect estimates of using modeled vs. monitored baseline air quality conditions in separate sensitivity analyses (Beig *et al* 2015) (see supplemental information section 2.4 and supplemental tables H and I). For more details on model configuration for the baseline year (2018), see section 2.6 and supplemental information section 1.3.5.

### 2.5. Modeling of 2030 scenarios

#### 2.5.1. Climate change drivers and meteorology

We estimated climate change impacts on temperatures in Ahmedabad using an existing data set corresponding to the Representative Concentration Pathway (RCP) 8.5 scenario, which indicated a change in the annual average temperature in Ahmedabad from 300.73 K in 2018 to 301.54 K in 2030 (an increase of 0.81 °C) (Monaghan *et al* 2014).

While increasing deployment of renewable energy makes it less likely that future global emissions will correspond to RCP 8.5, emissions trends in developing countries still track that high emissions trajectory (Pedersen *et al* 2020). Previous work integrating climate change, energy, and air quality modeling indicates that daily average temperature is an appropriate metric (Abel *et al* 2017). For more information on climate modeling methods, see supplemental information section 1.1.

#### 2.5.2. Future cooling energy demand

We previously analyzed the impacts of climate change-driven temperature increases, along with population and economic growth based on ICAP projections, on demand for electricity to cool buildings in Ahmedabad between 2018 and 2030 (Joshi *et al* 2022). That study estimated the annual electricity demand in Ahmedabad by 2030, the fraction of this 2030 electricity demand that would come from cooling demand in buildings, and the sensitivity of the modeled cooling demand to a range of future scenarios: climate change (the impact of rising temperatures due to climate change by 2030), mitigation (limiting the use of fossil fuels to meet additional cooling demand), and adaptation (implementing cool roofs to reduce on 2030 cooling demand). That prior analysis found that the climate change-triggered increase in 2030 cooling demand in Ahmedabad (0.17 terawatt-hours, TWh), a driver of cooling demand along with population growth and economic expansion, was slightly more than offset by an expansion of cool roofs from 5% to 20% of roof area in the city (energy savings of 0.21 TWh). The analysis described here applies key findings from that prior investigation to estimate 2030 air pollution and corresponding health impacts under different energy and land cover scenarios (BAU, M&A).

#### 2.5.3. 2030 business-as-usual (BAU) scenario overview

The prior Joshi *et al* (2022) analysis indicated that, under a BAU energy scenario, electricity supplied by the TPP would increase by approximately 6% between 2018 and 2030, absent any additional cool roof installations to moderate cooling energy demand. To estimate the air quality effects of this increase in power generation, we modified the emissions inventory deployed in WRF-Chem to increase direct air pollution emissions from the plant by a corresponding amount, and distributed changes in primary emissions to estimate air pollution concentrations in four representative months. The air pollution emission inputs impacted by power plant emissions are: (a) precursor gases: oxides of nitrogen and sulfur (NO*_X_*, SO_2_) from coal combustion, and (b) primary particulate matter e.g. black carbon from high temperature combustion, and primary PM_2.5_ (e.g. fly ash). For more information on energy modeling methods, see supplemental information section 1.2. For more information on selecting seasonally representative months analyzed in air pollution modeling, see section 2.6.1 and supplemental information section 1.3.5.

#### 2.5.4. 2030 mitigation and adaptation (M&A) scenario overview

##### 2.5.4.1. Mitigation

Recent emission inventories for Ahmedabad suggest that industrial sources, including thermal coal-fired power plants, contribute about 12.4% of observed PM_2.5_ concentrations in Ahmedabad (Ganguly *et al* 2020) and 9.8% in Gujarat overall (Cropper *et al* 2021). We estimated the municipal air quality effects of reducing 2030 emissions from TPP. With the NCAP goal of 20%–30% reductions in PM_2.5_ by 2024 (compared to 2017 levels) our modeling assumptions in table 1 represent plausible changes by 2030 (Ministry of Environment, Forests, and Climate Change (Government of India) 2019b). In the 2030 M&A scenario, we modify the underlying emissions inventory to completely remove the emissions from the coal plant, reflecting a shift of electricity supply away from fossil fuels and towards renewable energy (Joshi *et al* 2022). With our focus on identifying the air pollution and related health effects linked specifically to TPP emissions, we hold emissions for the other power plants depicted in figure 2 at their 2018 levels in boundary conditions for air modeling of both 2030 BAU and M&A.

This scenario is of interest because of policies that are transitioning India away from fossil fuel energy sources in alignment with India’s climate goals (United Nations Framework Convention on Climate Change 2015). In 2022, India’s Ministry of Power announced plans to reduce power generation at 81 coal-fired utilities because of availability of more cost-effective renewable energy (Varadhan 2022). While the country seeks to completely halt use of coal-fired power by 2070, the state of Gujarat has already announced it would stop granting permissions for new power plants in the state and indicated that renewable energy sources would be deployed to meet anticipated increases in energy demand (Vora 2019). These policies are already affecting operations at Ahmedabad’s TPP: in 2018, two units at station C of the plant (60 MW total) were decommissioned after more than 50 years of operation (Ministry of Power, Government of India 2018). The mitigation scenario we apply in this analysis (complete elimination of TPP emissions) helps to demonstrate the air quality and health effects linked with these national- and state-level policies. Our separate energy modeling analysis (Joshi *et al* 2022) indicates that by 2030, renewable energy supply to Ahmedabad will compensate for the energy supply reduction that would be caused by closure of TPP (see table 2) and that other plants that supply energy to Ahmedabad will continue to operate at full capacity (Joshi *et al* 2022).

**Table 2.** Results for climate and energy demand modeling, TWh denotes terawatt hours (sources: (Monaghan *et al* 2014, Joshi *et al* 2022 )).

	2018 baseline	2030 BAU	2030 M&A
Average temperature (°C)	27.58	28.39	28.39
Wind speed at 10 m (m s^−1^)	3.58	3.38	3.38
Relative humidity at 2 m (%)	55.13	53.10	53.10
Additional cool roof area coverage from 2018 (km^2^)	—	0	20.60
Torrent power plant (TPP) power supply to Ahmedabad (TWh)	1.70	2.10	0
Surat power supply to Ahmedabad (TWh)	4.43	5.26	5.26
Ahmedabad cooling electricity demand (TWh)	1.46	4.22	4.01
Renewable energy supply to Ahmedabad (TWh)	0.73	6.63	8.73

##### 2.5.4.2. Adaptation

To protect communities from extreme heat, Ahmedabad is implementing cool roofs to help keep indoor temperatures cooler in non-air-conditioned buildings. In a separate analysis (Joshi *et al* 2022), energy-demand impacts of expanding Ahmedabad’s municipal cool roof program to cover 20% of available roof area by 2030 were modeled, a substantial increase (i.e. an additional 15% of municipal roof area) beyond the current estimate of cool roofs covering approximately 5% of the city’s available roof area.

We applied an estimate for Ahmedabad of 14.2 kWh saved annually per square meter of cool roof coverage in our energy modeling for land cover adaptation (Bhatia *et al* 2011). Because we remove emissions from TPP coal combustion under the 2030 M&A scenario, the influence of cool roofs by 2030 reduces the cooling demand for electricity from regional renewable energy sources (taking into account growth in energy demand related to population growth and economic expansion, along with increasing temperatures).

Because the mitigation scenario assumes complete elimination of emissions from TPP, and the additional energy to meet cooling demand by 2030 assumed in our adaptation scenario would be supplied by non-polluting renewable energy (Joshi *et al* 2022), we combine these climate actions into a single 2030 M&A scenario. The main assumptions of the single baseline and two 2030 modeling scenarios described in sections 2.4 and 2.5 are summarized in table 1.

### 2.6. Co-benefits modeling inputs and outputs

#### 2.6.1. Air quality modeling

We modeled future air quality in Ahmedabad using the WRF-Chem model configuration for Ahmedabad specified by SAFAR (Beig *et al* 2021). The city air and health modeling grid domains are depicted in figure 3, along with the locations of the SAFAR air monitors and thermal coal-fired power plants in Ahmedabad and Gandhinagar. For 2018, meteorology is based on European Center for Medium-Range Weather Forecasts atmospheric reanalysis of the global climate, a meteorological dataset that has been validated for meteorological analyses in other settings (Kolluru *et al* 2020, Luo and Minnett 2020, Sharmar and Markina 2020, Jiang *et al* 2021, Tang *et al* 2021, Kannemadugu *et al* 2022). The model spin-up time is 10 d for each month; a detailed description of the SAFAR modeling framework is available in supplemental information section 1.3; model validation is documented elsewhere (Beig *et al* 2015, 2021).

For 2018 baseline modeling, we used chemical initial boundary conditions for gas and aerosols from Community Earth System Model (CESM) version 2.1/Community Atmosphere Model-Chem (Lamarque *et al* 2012) provided by the National Center for Atmospheric Research (NCAR), with a horizontal resolution 0.9° latitude × 1.25° longitude at 56 levels in the vertical domain available at six hourly intervals (National Center for Atmospheric Research n.d., Buchholz *et al* 2019). We applied NCAR CESM global bias-corrected CMIP5 output for the RCP 8.5 scenario (Monaghan *et al* 2014) to generate the 2030 initial boundary conditions for meteorological fields in WRF-Chem. Further details on air modeling inputs are described in supplemental information sections 1.3.3–4.

##### 2.6.1.1. Time horizon for future air quality modeling

Given constraints in computational resources and consistent with prior analyses, four months were selected in each modeled year, 2018 (baseline) and 2030 (future), to represent critical air pollution seasons: January (representing winter, December–February), May (representing summer/pre-monsoon, March–May), July (representing monsoon/rainy season, June–September), and October (representing post-monsoon/autumn, October–November). These seasonal period categorizations are consistent with those in prior published work by the Indian Institute of Tropical Meteorology, Indian Institute of Public Health-Gandhinagar, and the India Meteorological Department (Parthasarathy *et al* 1987, Sanjay *et al* 2020, Beig *et al* 2021, Wei *et al* 2021). Based on that seasonal representation, we calculated a manually-weighted daily city average PM_2.5_ level to characterize the annual concentration across the city. Our 2030 BAU and M&A scenarios modeled in WRF-Chem utilize the same selection of four months to enable comparison to the 2018 baseline scenario. We also estimated the health benefits from achieving three air quality targets by 2030 relative to the 2018 baseline: NCAP (reduction of PM_2.5_ pollution levels by 30% from baseline), NAAQS (40 *µ*g m^−3^), and WHO AQG (5 *µ*g m^−3^) (see supplemental information tables E and I).

#### 2.6.2. Health impact modeling

##### 2.6.2.1. Population and demographic data for modeling

To estimate population exposure to air pollution, the BenMAP-CE model applies population-based weighting to gridded WRF air pollution output (Sacks *et al* 2018). To account for population growth between 2018 and 2030, we developed a hybrid population dataset that incorporated the best available spatial resolution, population growth estimate, and age structure from several data sources ((United Nations Department of Economic and Social Affairs (Population Dynamics) 2018), (Ahmedabad Municipal Corporation n.d.), (US Environmental Protection Agency 2016) (Urban Emissions 2022)). See supplemental information section 1.4.1 and supplemental tables F and G for more information on our hybrid population estimation method and sensitivity analyses using different population datasets.

##### 2.6.2.2. Health metrics and baseline health estimates

Comparing the PM_2.5_ air pollution conditions for baseline 2018 versus those under two alternative future assumptions for 2030 (BAU and M&A), we quantify health co-benefits from avoided mortality using the BenMAP-CE model v1.5, an open-source software application used widely in health impacts research (Sacks *et al* 2018, Brown *et al* 2020, Manojkumar and Srimuruganandam 2021). Due to the lack of cause-specific mortality and morbidity data for Ahmedabad, we utilize all-cause mortality as the health metric in this study. All-cause mortality data were obtained from the Ahmedabad Municipal Corporation Office of the Registrar of Births and Deaths department.

##### 2.6.2.3. PM_2.5_ exposure-response functions

We calibrated BenMAP-CE with the gridded Ahmedabad population estimate for 2030 (see section 2.6.2). Due to the lack of India-specific exposure-response functions for all-cause mortality (Gordon *et al* 2018, Limaye *et al* 2018b), we applied integrated exposure-response functions for all-cause and non-accidental mortality in BenMAP-CE related to annual PM_2.5_ exposures (Pope *et al* 2015, Turner *et al* 2016, Burnett *et al* 2018) (see supplemental information section 1.4.3). The Burnett *et al* (2018) Global Exposure Mortality Model (GEMM), in particular, integrates data from 41 population cohorts in 16 countries with annual PM_2.5_ exposure levels up to 84 *µ*g m^−3^ (in China) (Yin *et al* 2018). The GEMM approach has previously indicated substantial health benefits from PM_2.5_ reductions in highly polluted settings including India and China (Burnett *et al* 2018).

## 3. Results

### 3.1. Climate change and cooling energy demand

Table 2 shows the results of our climate and energy demand modeling for Ahmedabad, extracted from a broader analysis (Joshi *et al* 2022). Climate warming causes the average temperature in the city to rise to 28.39 °C in 2030 from 27.58 °C in 2018, an increase of 0.81 °C (Monaghan *et al* 2014). Based on the analysis detailed in (Joshi *et al* 2022), annual demand for cooling electricity increases to 4.22 (BAU) TWh in Ahmedabad by 2030, compared to 1.46 TWh at 2018 baseline. But implementation of cool roofs to cover a total of 20% of available roof area in buildings can help to moderate this increase and reduce total annual electricity demand by 0.21 TWh. In our analysis of power sector emissions from TPP to help meet city electricity demand (Joshi *et al* 2022), we estimate an increase in total power generation (and corresponding primary pollution emissions) between 2018 and 2030 BAU (from 1.70 to 2.10 TWh) while power generation from Surat to help meet Ahmedabad’s demand increases from 4.43 to 5.26 TWh between 2018 and 2030. In our 2030 M&A scenario, we estimate zero electricity generation from TPP (in table 2, the share of 2030 BAU power supply from TPP, 2.10 TWh, is substituted by renewable energy in 2030 M&A) and eliminate the entirety of its pollution emissions for the purposes of air quality modeling.

Separately, our energy demand analysis reported that the cool roof intervention scenario and its associated reduction in electricity demand is equivalent to avoiding about 0.191 metric megatons of carbon dioxide emissions (Joshi *et al* 2022). The carbon dioxide reduction associated with the 2030 mitigation scenario is about ten times larger: between 1.97 and 2.61 metric megatons annually (see supplemental information section 2.2 and supplemental table C).

### 3.2. Air quality impacts

**Figure 4.**
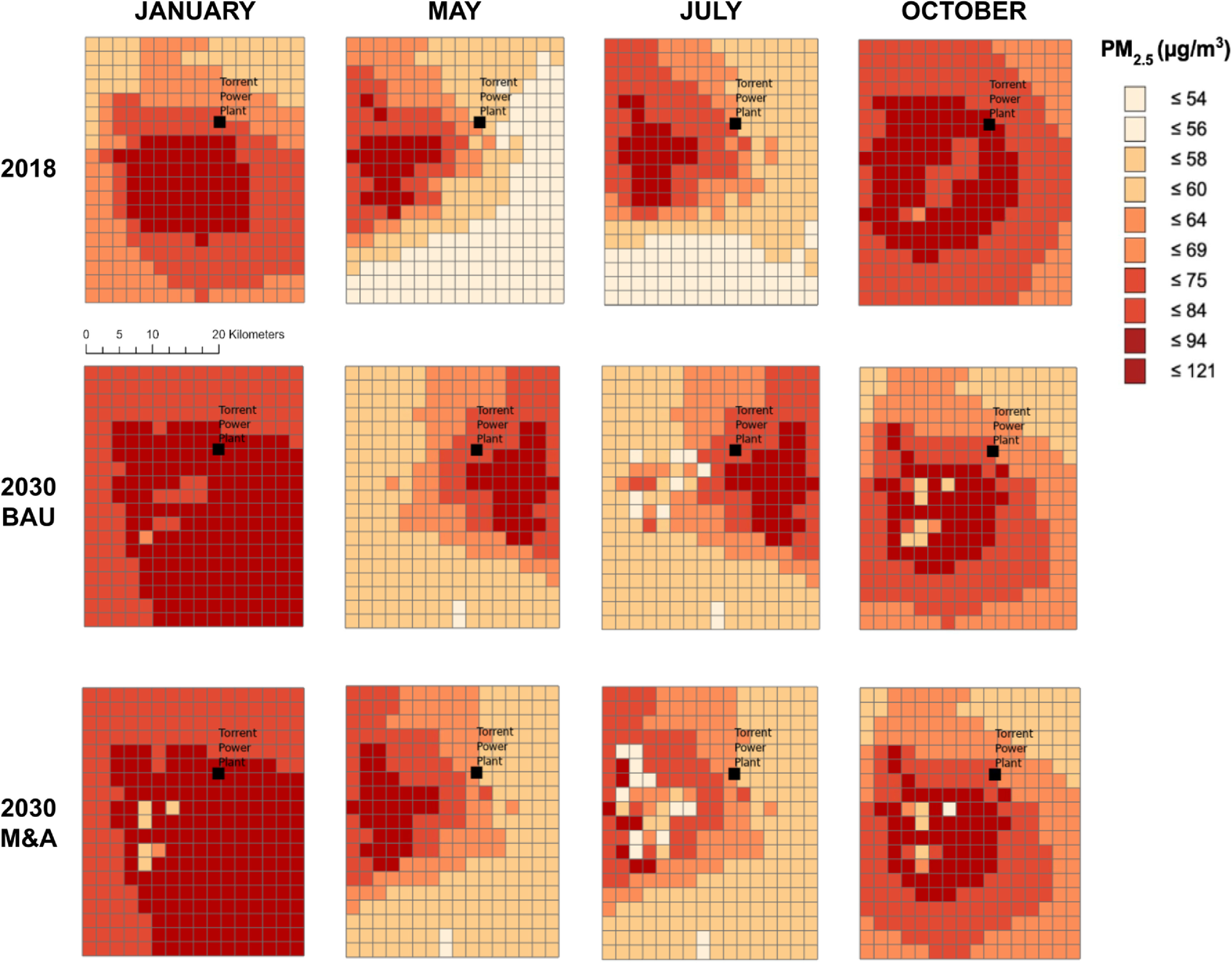
Monthly average PM_2.5_ estimates calculated from daily averages in WRF-Chem for the 2018 baseline, 2030 business-as-usual (BAU), and 2030 mitigation and adaptation (M&A scenarios). Comparing modeled 2030 vs 2018 air quality conditions, under different scenarios. Torrent power plant (TPP) (i.e. the Ahmedabad plant) location depicted with black square in each panel. Figure produced using ArcGIS Pro (ESRI 2020).

For air quality, we first describe our monthly results that represent four seasons: winter, spring, summer/monsoon and fall/post-monsoon. Figure 4 displays the WRF-Chem air quality modeling results, depicting daily PM_2.5_ levels averaged for each month under 2018 baseline, 2030 BAU, and 2030 M&A. The monthly average and standard distribution of PM_2.5_ levels for all grids for each scenario are shown in table 3.

**Table 3.** Monthly average PM_2.5_ estimates and standard deviations calculated from daily averages in WRF-Chem for the 2018 baseline, 2030 business-as-usual (BAU), and 2030 mitigation and adaptation (M&A) scenarios.

	Monthly average PM_2.5_ (*µ*g m^−3^) (standard deviation)			
Scenario	January	May	July	October
2018 baseline	74.46 (15.56)	69.72 (13.64)	38.95 (9.35)	86.20 (21.56)
2030 BAU	92.83 (14.40)	66.51 (12.61)	39.52 (8.23)	75.15 (17.14)
2030 M&A	92.42 (14.19)	66.37 (12.43)	38.13 (7.67)	74.95 (17.04)

Overall, across the three scenarios we identify consistent seasonal trends in air pollution, with January and October registering the highest levels and July registering the lowest levels. For annual weighting (described in supplementary section 1.3.5), the July data most strongly drive the annual average, but PM_2.5_ levels modeled for July are consistent across 2018, 2030 BAU, and 2030 M&A. Air pollution estimates for January show the highest increase from 2018 to both 2030 scenarios, while the October estimates show a decrease from 2018 to both 2030 scenarios. In our annual estimates, January data are weighted more heavily than October; that influence is apparent in our manually-weighted annual averages shown in table 4. It is important to note that for health impact estimates, BenMAP calculates population-weighted averages from the annual PM_2.5_ data we input (see table 4).

**Table 4.** BenMAP-CE input values and health result estimates in 2030 under BAU (business-as-usual) and M&A (mitigation and adaptation) scenarios. The 95% CI denotes lower and upper bounds of 95% confidence interval.

	2018 baseline	2030 BAU	2030 M&A
**BenMAP-CE inputs**			
Population (age 0–99)	8 459 139	9 308 479	9 308 479
Population (age 25–99)	6 344 354	7 488 672	7 488 672
Population (age 30–99)	5 921 397	7 116 333	7 116 333
Baseline mortality rate (All-cause, age 30–99, per 100 000)	684.21	—	—
Annual daily average PM_2.5_ (*µ*g m^−3^, manually weighted)	63.40	65.50	64.90
Annual daily average PM_2.5_ (*µ*g m^−3^, population weighted)	71.04	75.18	70.93
Change in PM_2.5_ (% change) from 2030 to 2018 (*µ*g m^−3^, population weighted annual average)	—	+4.13 (+5.81%)	−0.11 (−0.15%)
**BenMAP-CE health results**			
2030 excess annual mortality incidence, relative to 2018 baseline			
**Pope *et al* 2015** *All-cause mortality (95% CI)*	—	+1389 (1092, 1681)	−25 (−22, −28)
Average mortality rate (% change) (age 30–99, per 100 000)	—	707.95 (+3.47%)	697.89 (+1.02%)
**Turner *et al* 2016** *All-cause mortality (95% CI)*	—	+1193 (793, 1585)	−23 (−18, −27)
Average mortality rate (% change) (age 30–99, per 100 000)	—	704.60 (+2.98%)	690.20 (+0.88%)
**Burnett *et al* 2018** *Non-accidental mortality (95% CI)*	—	+870 (648, 1088)	−52 (−38, −66)
Average mortality rate (% change) (age 25–99, per 100 000)	—	699.06 (+2.17%)	689.27 (+0.74%)

Our results indicate that, by 2030, PM_2.5_ air pollution in Ahmedabad is expected to increase in the BAU climate scenario, but M&A actions can help to moderate this effect. The application of ambient meteorology, emissions, and atmospheric chemistry datasets through WRF-Chem modeling at a high spatial-resolution of 4.2 km^2^ provides more granular insights into local PM_2.5_ levels. Air quality improves slightly in the vicinity of TPP even in 2030 BAU due to differences in meteorology inputs between 2018 and 2030 and meteorology effects on secondary PM_2.5_ formation downwind of TPP (Pan *et al* 2021); eliminating emissions from TPP in 2030 M&A results in larger and more widespread PM_2.5_ reductions than those attributed to changing meteorology.

To assess how well our modeled 2018 air quality represents air quality observations from monitoring stations, we compared modeled and monitored data across four seasons (winter, spring, summer, and fall) (see supplemental information section 2.3). This comparison indicates that, during the fall season, the WRF-Chem Model well-represented PM_2.5_ concentrations (0.5% difference between measured and modeled PM_2.5_). However, during the other seasons the model underestimated PM_2.5_ concentrations by 20%–34.2% on average. Because the health impact analysis relies on the difference in pollution levels between the 2030 scenarios and 2018 baseline, any seasonal underestimation of PM_2.5_ by WRF-Chem that is systematic in both 2018 and 2030 should not affect the relative comparisons of associated premature mortality across different years.

### 3.3. Health impacts

**Figure 5.**
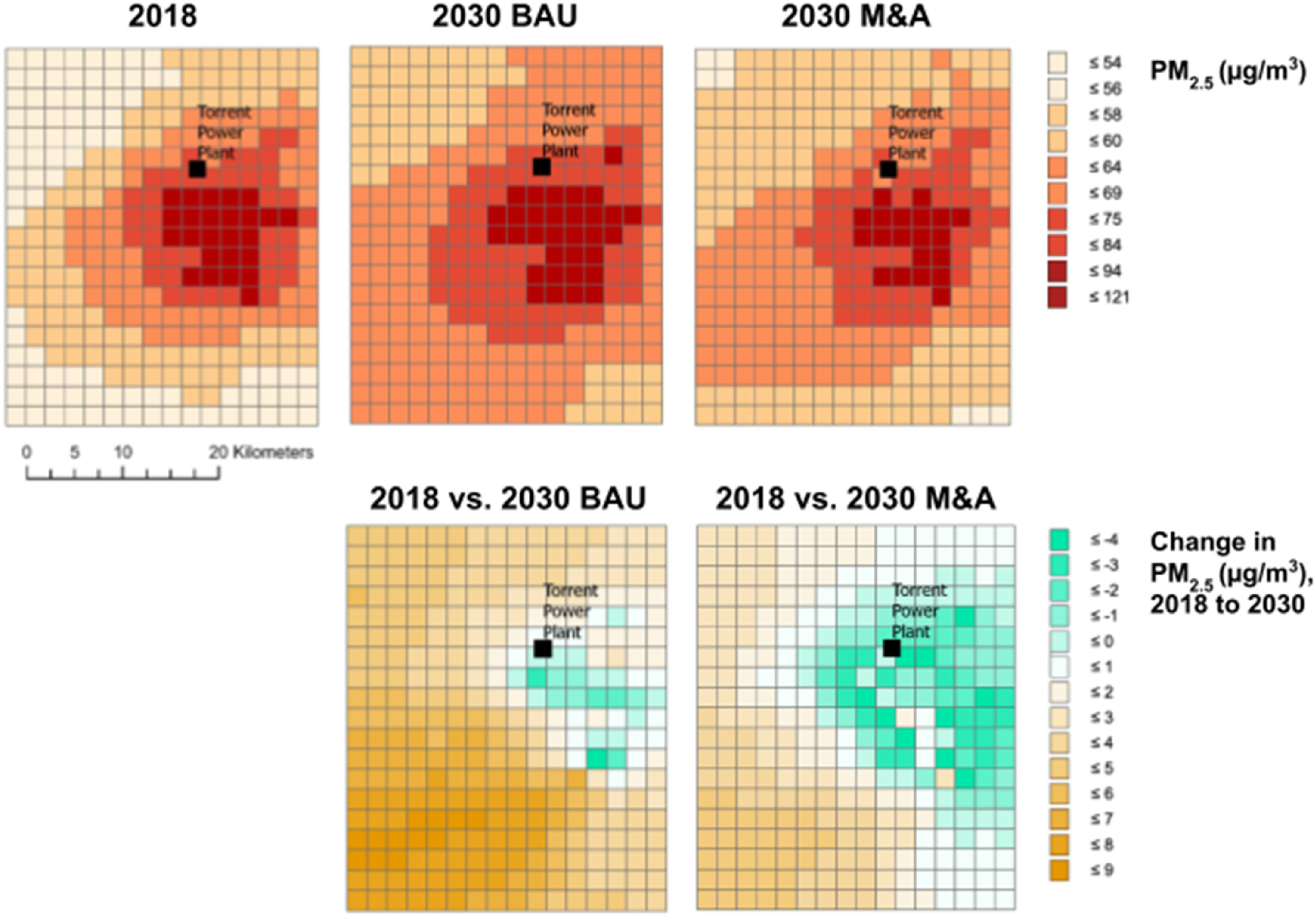
Comparing modeled 2030 vs. 2018 air quality conditions, under different scenarios. Top panels: annual daily average PM_2.5_ levels in study region (2018 baseline, 2030 business-as-usual (BAU) scenario, 2030 mitigation and adaptation (M&A) scenario). Bottom panels: changes in annual daily average PM_2.5_ relative to 2018 baseline (BAU: business-as-usual scenario; M&A: mitigation and adaptation scenario). In bottom panels, negative values (green tones) reflect lower air pollution concentrations in 2030 relative to 2018, and positive values (shown in orange tones) reflect higher air pollution concentrations in 2030 relative to 2018. Torrent power plant (TPP) (i.e. the Ahmedabad plant) location depicted with black square in each panel. Modeling grid at 4.2 km^2^ cell resolution, visualized in BenMAP-CE, reflects WRF-Chem modeled PM_2.5_ levels used as BenMAP-CE inputs. Figure produced using ArcGIS Pro (ESRI 2020).

In considering the exposed population, our analysis indicates that the overall population in the study region will increase by about 10% in 2030, from 8.46 million in 2018 to 9.31 million. As table 4 and figure 5 show, annual population-weighted daily average PM_2.5_ decreases by 0.11 *µ*g m^−3^ in 2030 relative to the 2018 baseline (a 0.2% decrease from baseline) in the M&A scenario, compared to a 4.13 *µ*g m^−3^ increase in the BAU scenario (a 5.8% increase from baseline). Population-weighted PM_2.5_ estimates derived via BenMAP-CE from WRF-Chem data (section 2.6.2) are considerably higher than manually-weighted WRF-Chem estimates for 2018, 2030 BAU, and 2030 M&A, demonstrating the importance of population data in BenMAP-CE to estimate exposure levels.

Our health effect estimates are for changes in excess annual all-cause mortality (age 30–99) and non-accidental mortality (age 25–99). We estimate that under the BAU scenario, the 2030 annual population-weighted PM_2.5_ level is associated with between 1193 (95% CI: 793–1585) and 1389 (95% CI: 1092–1681) excess all-cause deaths or 870 (95% CI: 648–1088) excess non-accidental deaths. However, we estimate reduced mortality in the 2030 M&A scenario relative to pollution-associated mortality the 2018 baseline. We estimate that under the M&A scenario, 2030 annual population-weighted PM_2.5_ is associated with 23 (95% CI: 18–27) to 25 (95% CI: 22–28) fewer all-cause deaths or 52 (95% CI: 38–66) fewer non-accidental deaths relative to the 2018 baseline. While the average all-cause mortality rate in people aged 30–99 rises in both 2030 scenarios across all exposure-response functions from 2018, the percent increase in the average mortality rate between 2018 and 2030 is about three-fold larger under BAU compared to the mortality rate under the M&A scenario.

Comparing between the two 2030 scenarios, we estimate that the reduced PM_2.5_ air pollution estimated in the 2030 climate action (M&A) scenario results in between 1216 and 1414 fewer all-cause deaths on average, or 922 fewer non-accidental deaths relative to the 2030 BAU scenario. Using the Burnett *et al* (2018) exposure-response function, the average mortality rate increases by 2.17% under 2030 BAU and decreases by about 0.74% under 2030 M&A, relative to the 2018 baseline. In our sensitivity analyses (reported in supplemental table E), we find that achievement of NCAP, NAAQS, or WHO AQG targets for annual daily average PM_2.5_ in 2030 would result in up to 6510, 9047, or 17 369 fewer premature all-cause deaths annually, respectively, relative to 2030 BAU.

## 4. Discussion

Our research, deploying an air pollution health impact assessment model for the first time in the Indian city of Ahmedabad, demonstrates that local actions to respond to climate change through M&A policies can achieve substantial air quality and health co-benefits at the local level. Local government, community and civil society groups are in discussion on the future of local power sources and plan to engage further given the Indian government’s aim to reduce output from coal-fired power plants. Efforts to fully substitute renewable energy in place of coal combustion in Ahmedabad are expected to improve local air quality relative to a BAU scenario. In contrast, a BAU scenario results in elevated emissions, PM_2.5_ concentrations, and higher all-cause mortality in 2030. For context, research by Vohra *et al* (2022) estimates that 2018 PM_2.5_ pollution from all sources in Ahmedabad caused 18 400 premature all-cause deaths (95% CI: 5400–31 400) (Vohra *et al* 2022); the 2030 health benefits achievable in our M&A scenario (compared to BAU) therefore represent an approximate 7% reduction in PM_2.5_-related mortality at the city level. The baseline 2018 annual population-weighted average PM_2.5_ level estimated in our WRF-Chem modeling (71.04 *µ*g m^−3^) is higher than a recently published estimate 57.68 *µ*g m^−3^ for 2019 (an exposure level corresponding in that same study to an estimated 5960 premature deaths) (Health Effects Institute 2022); these divergent findings indicate the need for further refinement of air pollution exposure and health effect estimates in Ahmedabad and India overall.

This study takes advantage of newly-established air quality monitoring and forecasting in Ahmedabad to enable novel insight into the local air quality and health co-benefits of climate actions over the near-term. Future projections of climate change impacts on heat, cooling demand, air pollution, and human health are needed at a local level in India. A key challenge in quantifying the air quality and health co-benefits of M&A actions is the range of data, compatible modeling platforms, and expertise needed to conduct comprehensive, integrated analyses; our interdisciplinary research team surmounted this obstacle by linking climate, energy, air quality, and health datasets and models using consistent methods that account for not just future projected climate change, but also population growth and economic expansion.

Several data issues and modeling assumptions could limit further application of our analyses. For our air quality modeling, due to computational constraints we did not undertake a year-round, meteorology-constant simulation of 2030 BAU and 2030 M&A using 2018 meteorology inputs; nor run a modeling ensemble method across a range of future years to thoroughly assess the effects of climate change on meteorology and air quality. As a result, we cannot determine the portion of air pollution change between 2018 and 2030 due to changing meteorology alone. The project team did not have access to cause-specific or age-specific daily mortality data for Ahmedabad, nor to morbidity data and we were unable to apply local, age-specific exposure-response coefficients to estimate the potential health co-benefits among residents <25 years. Because children are especially vulnerable to air pollution health harms (Perera *et al* 2019, deSouza *et al* 2022), the avoided mortality co-benefits will therefore be underestimated. In our 2030 M&A scenario, we only considered emissions reductions from a single stationary source rather than additional stationary and mobile air pollution sources. While prior analyses indicate that renewable energy sources are poised to grow substantially by 2030, our air quality and health modeling assumes that these sources will help to substitute for reduced power generation from TPP along with continuing power generation at the two Surat plants.

With regards to the mitigation scenario we considered in our modeling, targets announced in 2021 would allow for India to provide half the country’s energy mix from renewable, non-fossil fuel sources by 2030 (United Nations Framework Convention on Climate Change 2021). With India’s climate change policy ambitions in mind, our approach provides an adaptable method to estimate local air pollution and health co-benefits of renewable energy deployment and passive cooling strategies in buildings. Cities need more information on the health benefits of reduced climate and air pollution (Bruine de Bruin *et al* 2021). With M&A projects already underway in Ahmedabad and other Indian cities, our modeling results on substantial avoidable premature mortality from air quality improvements strengthen the evidence base to scale up climate solutions throughout India.

## Data Availability

All data that support the findings of this study are included within the article (and any supplementary information files).
